# In reply to the letter to the editor from Jin et al: critical insights into the role of miR-290 and miR-302 clusters in iPSC reprogramming

**DOI:** 10.1093/stmcls/sxaf046

**Published:** 2025-09-05

**Authors:** Robert Blelloch

**Affiliations:** Eli and Edythe Broad Center of Regeneration Medicine and Stem Cell Research, University of California at San Francisco, San Francisco, CA 94143, USA; Center for Reproductive Sciences, University of California at San Francisco, San Francisco, CA 94143, USA; Department of Urology, University of California at San Francisco, San Francisco, CA 94143, USA

**Keywords:** microRNAs, miR-290, miR-302, reprogramming, induced pluripotent stem cells, ESCC miRNAs, pluripotency

## Text

We thank the authors of this letter for their highly positive comments on our recent paper, “The miR-290 and miR-302 clusters are essential for reprogramming of fibroblasts to induced pluripotent stem cells.” We also concur with their insightful suggestions for further exploration. Specifically, they highlight 2 key areas of interest.

First, they raise the importance of learning the mRNA targets of these microRNAs that are crucial for enabling the induction of pluripotency. Each microRNA can potentially suppress hundreds of target mRNAs, and we believe it is the combination of these targets that underlies their role. Although this complexity makes dissecting the mechanism challenging, it also offers a pathway to uncovering critical suppression mechanisms acting as barriers to reprogramming to iPSCs, as demonstrated in previous publications of ours.^1-3^

Second, they inquire about the developmental potential of induced pluripotent stem cells (iPSCs) arising from the individual loss of either miR-290 or miR-302. In the current paper, we show that the loss of either cluster alone still permits reprogramming to iPSCs that are molecularly identical to ESCs lacking the corresponding microRNA cluster, whereas the loss of both clusters completely blocks reprogramming. Although the absence of either cluster alone allows for reprogramming to iPSCs, our previous findings indicate that their developmental potential will likely be altered, given that deletion of either cluster in ESCs affects their differentiation.^4-6^ We believe this reflects their role in differentiation rather than reprogramming, which is certainly a subject of substantial interest.
